# A molecular analysis of desiccation tolerance mechanisms in the anhydrobiotic nematode *Panagrolaimus superbus *using expressed sequenced tags

**DOI:** 10.1186/1756-0500-5-68

**Published:** 2012-01-26

**Authors:** Trevor Tyson, Georgina O'Mahony Zamora, Simon Wong, Máirin Skelton, Brian Daly, John T Jones, Eoin D Mulvihill, Benjamin Elsworth, Mark Phillips, Mark Blaxter, Ann M Burnell

**Affiliations:** 1 Department of Biology, National University of Ireland Maynooth, Maynooth, Co. Kildare, Ireland; 2 Irish Centre for High-End Computing, Trinity Technology & Enterprise Campus Grand Canal Quay, Dublin 2, Ireland; 3 Plant Pathology, The James Hutton Institute, Invergowrie, Dundee DD2 5DA, UK; 4 Institute of Evolutionary Biology, Ashworth Laboratories, King's Buildings, The University of Edinburgh, Edinburgh EH9 3JT, UK

## Abstract

**Background:**

Some organisms can survive extreme desiccation by entering into a state of suspended animation known as anhydrobiosis. *Panagrolaimus superbus *is a free-living anhydrobiotic nematode that can survive rapid environmental desiccation. The mechanisms that *P. superbus *uses to combat the potentially lethal effects of cellular dehydration may include the constitutive and inducible expression of protective molecules, along with behavioural and/or morphological adaptations that slow the rate of cellular water loss. In addition, inducible repair and revival programmes may also be required for successful rehydration and recovery from anhydrobiosis.

**Results:**

To identify constitutively expressed candidate anhydrobiotic genes we obtained 9,216 ESTs from an unstressed mixed stage population of *P. superbus*. We derived 4,009 unigenes from these ESTs. These unigene annotations and sequences can be accessed at http://www.nematodes.org/nembase4/species_info.php?species=PSC. We manually annotated a set of 187 constitutively expressed candidate anhydrobiotic genes from *P. superbus*. Notable among those is a putative lineage expansion of the *lea *(late embryogenesis abundant) gene family. The most abundantly expressed sequence was a member of the nematode specific *sxp/ral-2 *family that is highly expressed in parasitic nematodes and secreted onto the surface of the nematodes' cuticles. There were 2,059 novel unigenes (51.7% of the total), 149 of which are predicted to encode intrinsically disordered proteins lacking a fixed tertiary structure. One unigene may encode an exo-β-1,3-glucanase (GHF5 family), most similar to a sequence from *Phytophthora infestans*. GHF5 enzymes have been reported from several species of plant parasitic nematodes, with horizontal gene transfer (HGT) from bacteria proposed to explain their evolutionary origin. This *P. superbus *sequence represents another possible HGT event within the Nematoda. The expression of five of the 19 putative stress response genes tested was upregulated in response to desiccation. These were the antioxidants *glutathione peroxidase, dj-1 *and *1-Cys peroxiredoxin*, an *shsp *sequence and an *lea *gene.

**Conclusions:**

*P. superbus *appears to utilise a strategy of combined constitutive and inducible gene expression in preparation for entry into anhydrobiosis. The apparent lineage expansion of *lea *genes, together with their constitutive and inducible expression, suggests that LEA3 proteins are important components of the anhydrobiotic protection repertoire of *P. superbus*.

## Background

Dehydration is a severe stress for organisms--most animals die if they lose more than 15-20% of their body water [1], while loss of more than 20-50% of their water content is lethal to most higher plants [2]. Some organisms have the capacity to survive extreme desiccation by entering into a state of suspended animation known as anhydrobiosis [3]. When rehydrated, anhydrobiotes revive and resume active metabolism. For example, a viable culture of the nematode *Panagrolaimus *sp. PS443 was isolated from dry soil that had been stored for 8 years [4]. An understanding of the molecular mechanisms responsible for anhydrobiotic survival will provide insights which may ultimately lead to the ability to confer desiccation tolerance on desiccation sensitive organisms by utilizing the strategies of anhydrobiosis, a development termed anhydrobiotic engineering [5]. Anhydrobiotic taxa have a wide distribution in nature, being found in bacteria, archaea, fungi, invertebrates, terrestrial microalgae, mosses, lichens, plant seeds and pollen, and there are approximately 350 species of angiosperm "resurrection plants" [6]. This distribution demonstrates that anhydrobitoic phenotypes are likely to have evolved independently on multiple occasions and provides support for the concept of anhydrobiotic engineering. Invertebrate anhydrobiotes include members of the Nematoda, Rotifera, Tardigrada, Crustacea and Insecta. These anhydrobiotes typically occupy aquatic or terrestrial habitats that are prone to temporary water loss. Free-living nematodes, rotifers and tardigrades contain representatives which are capable of entering anhydrobiosis at all stages of their life cycle. Crustacean anhydrobiotic stages are confined to the embryonic cysts of aquatic brine shrimps and other microcrustaceans [7]. The chironomid *Polypedilum vanderplanki *is the only anhydrobiotic insect described to date, but an anhydrobiotic capacity is restricted to the aquatic larval stages of this insect [8].

Most anhydrobiotic organisms are slow dehydration strategists [9], being unable to survive exposure to extreme desiccation unless they have first experienced a period of gradual water loss at high relative humidity (RH). During this period of slow dehydration the biochemical and molecular changes necessary for anhydrobiotic survival are induced. Slow dehydration strategists are found in aquatic and terrestrial habitats that lose water slowly. Many lichens, algae and bryophytes can survive rapid water loss; their tissues can tolerate the passage from the fully hydrated state to air dryness within an hour [10]. The bryophyte *Tortula ruralis *can survive rapid cellular dehydration and this vegetative desiccation tolerance is characterised by two components: constitutive expression of protective molecules and an inducible repair and recovery programme that is activated upon rehydration [10,11]. Some nematodes which live in exposed environments such as moss cushions, or the aerial parts of plants can also survive rapid desiccation [9,12]. Perry and Moens have recently proposed that, for nematode anhydrobiotes, the terms slow- and fast-dehydration strategists be replaced by external dehydration strategists and innate dehydration strategists, respectively [13]. External dehydration strategists, having little independent ability to control water loss, occur in environments that experience slow rates of water loss; whereas innate dehydration strategists have intrinsic adaptations to control the rate of water loss. These intrinsic adaptations may include behavioural (coiling/clumping) responses or morphological adaptations (e.g. surface lipids [14]) that slow the rate of water loss and allow time for inducible molecular protection mechanisms to be put in place. It may also be possible that, like the bryophyte *T. ruralis*, some innate dehydration strategist nematodes may also be constitutively adapted at a cellular level to survive desiccation [15].

**Figure 1:**
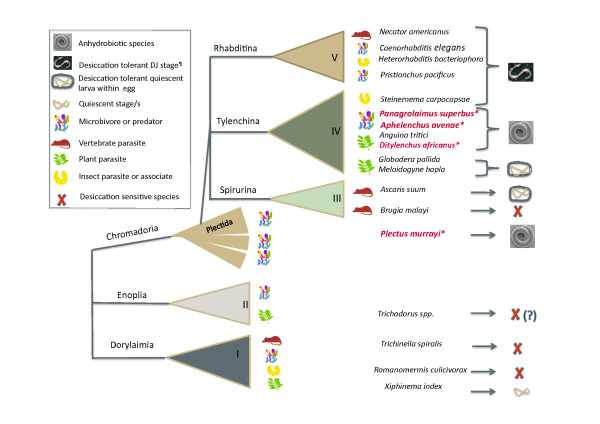
**A phylogenetic tree of the Phylum Nematoda based on that of Blaxter *et al***. [**25**]**showing the distribution of anhydrobiotic and desiccation tolerant taxa, along with their trophic ecology, across the Phylum**. (¶Erkut *et al. *have recently shown that *C. elegans *dauer larvae can survive exposure to low relative humidity following preconditioning at 98% RH for 4 days [26]).

Nematodes are a species-rich phylum with members occurring in marine and freshwater sediments, in soil and in moist terrestrial habitats. The phylum also contains economically important parasites of plants and animals. Free living anhydrobiotic nematodes occur in habitats susceptible to desiccation, but a capacity to undergo anhydrobiosis has also been important in the evolution of parasitic nematodes since many parasitic nematodes have anhydrobiotic infective stages or cysts [16] (Figure 1). Anhydrobiotic nematodes are abundant in the sub-order Tylenchina, a diverse group of nematodes that contains free living microvores and predators, as well as parasites of invertebrates, vertebrates and plants. The Tylenchina are particularly noted for the repeated evolution of plant parasitic clades within this sub-order [17] and phylogenetic reconstructions show that anhydrobiotic Tylinchina also have had multiple independent evolutionary origins. The genus *Panagrolaimus*, a member of the Tylenchina, contains both external dehydration and innate dehydration strategist nematodes [15,18,19]. Panagrolaimids feed on bacteria and occupy a variety of niches ranging from Antarctic, temperate and semi-arid soils to terrestrial mosses. *Panagrolaimus *species have a short generation time (~ 10 days at 20°C); they can be cultured with *Escherichia coli *as a food source using protocols developed for the model nematode *Caenorhabditis elegans *and an RNA interference protocol has been described for *P. superbus *[20]. In addition to its anhydrobiotic capacity, *P. davidi *from maritime Antarctica can survive freezing when fully hydrated [21]. We have found that a similar cryotolerant capacity also exists in other hydrated *Panagrolaimus *species isolated in temperate and continental regions (McGill *et al*., unpublished). Phylogenetic analysis shows that the anhydrobiotic species and strains of *Panagrolaimus *described to date belong to a single clade [15], which will facilitate comparative transcriptome analyses of the molecular basis of dehydration within a single genus. Here we present an analysis of 4,009 unique sequences (unigenes) derived from 9,216 Sanger-sequenced ESTs from an unstressed mixed population of the innate/fast desiccation strategist *Panagrolaimus superbus *and we identify putative anhydrobiosis related genes which may have application in future anhydrobiotic engineering experiments. *P. superbus *was originally isolated in 1981 from a gull's nest in Surtsey [22], an Icelandic island formed during 1963-1967 from volcanic eruptions [23]. The objective of the 959 Nematode Genomes Initiative is to encourage genome sequencing across the diversity of the phylum Nematoda [24]. Because of its provenance and its anhydrobiotic and cryotolerant phenotypes the genome of *P. superbus *is currently being sequenced as part of the Nematode Genomes Initiative (http://www.nematodes.org/nematodegenomes/index.php?title=About#tab=Welcome). In addition to providing cDNA clones and sequence data for candidate anhydrobiotic genes, the dataset presented here will also provide anchor sequences important for the assembly of the genome and transcriptome of *P. superbus*.

## Results and discussion

### EST assembly

A total of 9,216 ESTs were obtained by Sanger sequencing from a directionally cloned cDNA library prepared from an unstressed mixed stage population of *P. superbus*. Processing these ESTs through the PartiGene pipeline [27] resulted in 7,606 high quality ESTs with an average length of 425 bp (Table 1). These EST sequences have been deposited in dbEST with the accession numbers GW405912-GW413517. The Partigene pipeline then clustered these ESTs into 1,079 consensus sequences (contigs) and 2,958 singletons. Removal of putative bacterial sequences and rRNA genes yielded a total of 3,982 putative protein-coding transcripts (unigenes). BLASTX analysis showed that 1,923 of these unigenes had significant hits to a combined NemPep+, WormPep and the NCBI non-redundant (nr) NCBI database (see Methods for details). Among these were 100 unigenes which had unique hits to the NCBI nr database, leaving 2,059 (51.7%) novel *P. superbus *unigenes. These *P. superbus *unigene annotations can be subjected to keyword queries and the sequences can be downloaded from the NEMBASE4 database at http://www.nematodes.org/nembase4/species_info.php?species=PSC.

**Table 1:** Summary of the analysis of expressed sequence tag (EST) sequences from a cDNA library prepared from a mixed stage unstressed culture of the fast desiccation strategist nematode Panagrolaimus superbus

Number of raw sequences	9,216
Number of high quality sequences	**7,606**
Average length of high quality EST sequences	425 ± 193
Total number of contigs	1,079
Total number of singletons	2,958
Number of putative bacterial contaminant sequences	28
Number of rRNA gene consensus sequences	27
Number of mtDNA consensus sequences	10
Number of putative unigenes (excluding bacterial contaminants and rRNA genes, but including the mtDNA genes)	**3,982**
Number of unigenes with significant hits^1 ^to the NemPep4^+2^, WormPep^3 ^and NR^4 ^combined database	1,923
Number of unigenes with unique hits to the NR database, but not to NemPep4 or WormPep	100
Number of unigenes with no significant BLAST hits	**2,059 **(51.7%)

^1^Blast cutoff value was 1e^-4^

^2^http://www.nematodes.org/

^3^http://www.sanger.ac.uk/Projects/C_elegans/WORMBASE/current/wormpep.shtml

^4^http://www.ncbi.nlm.nih.gov/blast/blast_databases.shtml

Of the 100 unigenes with unique hits to the NCBI nr database, 38 returned BLAST hits only to bacterial taxa. Bacterial sequences had been screened and removed from the dataset during the assembly process using stringent matching criteria (BLASTN with an e-value cut-off of 1e^-50^), thus some of these 38 sequences may correspond to residual contaminant sequences. Others may represent sequences from bacterial associates of *P. superbus*, or horizontally transferred sequences, e.g. one unigene sequence (PSC01785) had highest similarity to an ankyrin gene from a *Wolbachia *endosymbiont from *Culex quinquefasciatus *[28]. The remaining 62 sequences returned hits to eukaryote taxa and these included several hits to plant LEA (late embryogenesis abundant) sequences, important in plant desiccation tolerance. PSC01785 had best similarity to an exo-β-1,3-glucanase, a member of the glycosyl hydrolase family 5 (GHF5), from the Stramenopile Oomycete *Phythophthora infestans *[29]. β-1,3-glucans are a major structural component of fungal cell walls and, as a microbivore, *P. superbus *may also use GH5 glucanases to incorporate soil fungi into its diet. Endo-β-1,3-glucanase GHF5 genes have been reported from the fungal feeding nematodes *Bursaphelenchus xylophilus, B. mucronatus *[30] and *Aphelenchus avenae *[31], with horizontal gene transfer (HGT) from bacteria being proposed to explain their evolutionary origin. Endo-β-1,4-glucanase (cellulase) genes, also belonging to GHF5, have been reported from several species of plant parasitic nematodes of the order Tylenchida [32], with HGT from bacteria also proposed as their likely source [33]. The GHF5 cellulases recently reported from the free living nematode *Pristionchus *[34] are most similar to sequences from two slime mould species (Amoebozoa). Thus the *P. superbus *sequence reported here represents another possible HGT event (this time from the eukaryote Stramenopile lineage) for nematode GHF5 genes.

Large EST datasets have been generated for nematode parasites, however with the exception of the model nematodes *C. elegans*, other *Caenorhabditis *species and *Pristionchus pacificus*, there are relatively few EST datasets for free-living nematodes. Metagenomic analyses of nematode ESTs [35,36] show that nematode gene-space is substantially under-sampled. BLAST analyses of the EST cluster consensus sequences from 37 species represented in NemPep3 showed that 34-70% of the sequences from each nematode species were unique to that species [35]. Data from Nembase4 (derived from 54 parasites and 8 free living species) show that 61.8% of the predicted proteins had no GO, EC or KEGG annotations [37]. Thus the finding that 51.7% of the *P. superbus *unigenes correspond to novel sequences is consistent with previous studies and is a reflection of the diversity of nematode gene space.

### Abundantly expressed transcripts

The 44 most abundantly expressed *P. superbus *protein-coding sequences comprise 1,200 ESTs and represent 15.7% of the total EST dataset (Table 2). The most abundant sequence, containing 79 ESTs, encodes a member of the nematode specific family of SXP/RAL-2 proteins [38,39]. These proteins have been detected in the pharyngeal glands and as secreted and surface associated antigens in diverse animal parasitic nematodes. Immunization with recombinant antigens derived from SXP/RAL-2 has been effective in protecting treated animal hosts against filarial worm [40], roundworm [41] and hookworm [42] infections. SXP/RAL-2 proteins have also been described in plant parasitic nematodes [43,44] and SXP/RAL-2 sequences from 11 species of plant parasitic nematodes are represented in NEMBASE4. GenBank searches show that SXP/RAL-2 homologs also occur in other free living nematodes in addition to *P. superbus *viz.: *C. elegans *(Accession Numbers: NP_496220 and NP_495640); *C. briggsae *(XP_002630517) and *Pristionchus pacificus *(5 ESTs). Nematode SXP/RAL-2 sequences are likely to be encoded by a small multigene family [43,44]. We detected five SXP/RAL-2 unigenes in *P. superbus*, comprising 95 ESTs and representing 2.2% of the total EST dataset. With the exception of *Ascaris lumbricoides*, the level of SXP/RAL-2 expression in *P. superbus *was higher than that observed for any of the 19 species of parasites with SXP/RAL-2 homologs in NEMBASE4. SXP/RAL-2 are small (16-21 kDa) basic proteins which share a common domain of unknown function (DUF148, PF02520 http://pfam.sanger.ac.uk/). No RNAi phenotypes have been detected for SXP/RAL-2 homologs in *C. elegans*, but one homolog (NP_496220/ZK970.7) was among 14 genes which were upregulated in *C. elegans *on response to fungal infection [45]. All SXP/RAL-2 sequences characterised to date, including PSC00077, have a signal peptide indicative of a secreted protein. Parasitic nematode studies suggest that these nematode-specific proteins are most likely secreted from the pharyngeal glands onto the surface of the cuticle where they appear to carry out a structural or protective function. The very high level of expression of SXP/RAL-2 sequences in *P. superbus *suggests that this cuticular protein may have an important role in anhydrobiotic protection in this nematode.

**Table 2:** Most abundantly represented transcripts in a dataset of 7,606 ESTs prepared from a mixed stage unstressed culture of the anhydrobiotic nematode *Panagrolaimus superbus*.

*Cluster ID*	*Blast Identity [Organism]*	Accession Number* ^§^	*BLAST Score & E value*		*Number of ESTs (Percentage of Total EST Dataset)*
PSC00077	Immunodominant antigen (SXP/RAL-2 protein) [*Ancylostoma caninum*]	ABD98404.1	304	3e^-26^	79 (1.04)
PSC00006	No significant similarity found	-	-	-	71 (0.93)
PSC00009	No significant similarity found	-	-	-	70 (0.92)
PSC00076	No significant similarity found	-	-	-	54 (0.71)
PSC00137	No significant similarity found	-	-	-	49 (0.64)
PSC00511	Major sperm protein [*Caenorhabditis elegans*]	NP_494858.1	635	1e^-64^	40 (0.53)
PSC00155	Hypothetical protein (Y105C5A.8) [*C. elegans*]	NP_001041003	430	1e^-40^	39 (0.51)
PSC00051	No significant similarity found	-	-	-	35 (0.46)
PSC01915	Major sperm protein [*C. elegans*]	NP_494858.1	640	3e^-65^	30 (0.39)
PSC00043	Yolk protein (vitellogenin) (*CEW1-vit-6*) [*Oscheius sp*.]	U35449	280	6e^-23^	29 (0.38)
PSC00163	No significant similarity found	-	-	-	29
PSC00182	No significant similarity found	-	-	-	29
PSC00025	No significant similarity found	-	-	-	28 (0.37)
PSC00165	Major sperm protein [*C. elegans*]	NP_494858.1	640	3e^-65^	27 (0.35)
PSC00883	No significant similarity found	-	-	-	27
PSC00633	No significant similarity found	-	-	-	26 (0.34)
PSC00187	Lysosomal protein (*heh-1*) [*C. elegans*]	NP_497671.2	295	3e^-25^	25 (0.33)
PSC00252	Expressed sequence tag [*Meloidogyne chitwoodi*]	MCP06382^§^	358	2e^-32^	25
PSC00203	No significant similarity found	-	-	-	25
PSC00316	No significant similarity found	-	-	-	25
PSC00610	No significant similarity found	-	-	-	24 (0.31)
PSC00167	Vitellogenin (*vit-5*) [*C. elegans*]	NP_508589	238	2e^-18^	23 (0.30)
PSC00241	No significant similarity found	-	-	-	22 (0.29)
PSC00429	No significant similarity found	-	-	-	22
PSC00179	Major sperm protein [*C. elegans*]	NP_501781.1	634	1e^-64^	21 (0.28)
PSC00876	Lipid binding protein (*lbp-3*) [*C. elegans*]	NP_001041249	360	9e^-33^	21
PSC00004	No significant similarity found	-	-	-	21
PSC00010	No significant similarity found	-	-	-	21
PSC00566	Major sperm protein [*C. elegans*]	NP_494898	633	2e^-64^	20 (0.26)
PSC00265	60S ribosomal protein L7a [*Loa loa*]	XP_003139379.1	883	e^-93^	19 (0.25)
PSC00457	Actin family member [*Panagrellus redivivus*]	AAM47606.1	1187	3e^-148^	19
PSC00015	Histone H4d [*Xenopus laevis*]	NP_001128541.1	469	2e^-45^	17 (0.22)
PSC00128	Cytochrome c oxidase subunit 1 [*Chabertia ovina*]	YP_003434131.1	1300	3e^-141^	17
PSC00326	Major sperm protein [*B. malayi*]	XP_001902608.1	210	2e^-15^	17
PSC00122	No significant similarity found	-	-	-	17
PSC00047	60S ribosomal protein L37a [*B. malayi*]	XP_001902009.1	416	3e^-39^	16 (0.21)
PSC00764	Expressed sequence tag [*M. chitwoodi*]	MCP06382_1^§^	522	4e^-51^	16
PSC00097	No significant similarity found	-	-	-	16
PSC00962	Expressed sequence tag[*Angiostrongylus cantonensis*]	AAC00593_1^§^	198	6e^-14^	15 (0.19)
PSC00184	Major sperm protein [*Dictyocaulus viviparus*]	ABW37697.1	624	2e^-63^	15
PSC00064	Eukaryotic elongation factor 1A [*B. xylophilus*]	ACZ13348.1	1,348	8e^-147^	15
PSC00486	Major sperm protein [*C. elegans*]	NP_494858.1	640	3e^-65^	15
PSC00127	No significant similarity found	-	-	-	15
PSC00725	No significant similarity found	-	-	-	15

The consensus sequence for each EST was translated using prot4EST [46] and was then subjected to a BLASTP search against a non-redundant custom database comprising NCBI (nr), NemPep + and WormPep sequences

* NCBI (nr) accession numbers are given when available

^§^Three unigenes had hits only to NemPep EST sequences from the NEMBASE4 database

Ten abundantly expressed sequences were associated with reproductive function, eight corresponding to major sperm protein genes (MSPs) and two to vitellogenin genes. In total we detected 32 *P. superbus *MSP unigenes and 7 vitellogenin unigenes. In *C. elegans *MSPs are encoded by a multigene family comprising more than 50 genes [47,48]. Nematode MSPs are small, basic proteins required for the amoeboid movement of sperm. A family of six genes *vit-1 *to *vit-6 *encode *C. elegans *vitellogenin [49,50], a major yolk component which is expressed exclusively in the adult hermaphrodite intestine from which it is secreted into the pseudocoelomic space and taken up by oocytes [51]. Four structural genes were abundantly expressed in *P. superbus*: an actin family member (homolog of *C. elegans act-2*); a gene encoding a core histone protein required for chromatin assembly and chromosome function [52] and genes encoding two proteins associated with the 60S ribosomal subunit. Two abundantly expressed contigs were associated with lipid metabolism. PSC00187 encodes a homolog of *C. elegans *HEH-1 and human NPC2/He1, a cholesterol-binding protein whose deficiency in humans causes Niemann-Pick type C2 disease involving retention of cholesterol in lysosome [53,54]. Transcripts for the mitochondrially encoded cytochrome c oxidase subunit 1, essential for oxidative phosphorylation and ATP synthesis, were also highly expressed in this mixed stage *P. superbus *library.

Twenty one of the abundant sequences listed in Table 2 are novel. These novel unigenes correspond to 641 ESTs, representing 8.4% of the total EST dataset. Data on the predicted physico-chemical parameters of the putative proteins encoded by these 21 unigenes are presented in Additional file 1. Thirteen (65%) of these novel unigenes encode a signal peptide indicative of a secreted protein. The association between sequence novelty and likely secretion has been noted previously in the parasitic nemataode *Nippostrongylus brasiliensis *[55]. Three of the putative novel proteins are predicted to be natively unfolded over 80-100% of their primary sequence. The *P. superbus *dataset contains a total of 2,059 novel unigenes. Further analysis of these novel sequences is presented in a later section.

### Functional annotation

In order to identify candidate stress-related genes which may have a role in anhydrobiosis the annot8r program [56] was used to assign KEGG (Kyoto Encyclopaedia of Genes and Genomes) pathway annotations [57] and Gene Ontology (GO) terms [58] to the *P. superbus *unigenes.

#### Assignments to metabolic pathways using KEGG

**Figure 2:**
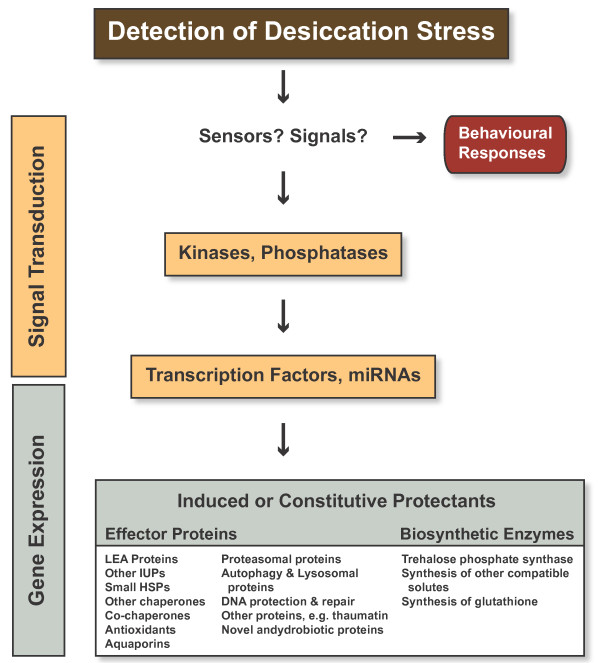
**Possible steps involved in the detection and expression of anhydrobiotic protection mechanisms in nematodes (sHSP = small heat shock protein; LEA = late embryogenesis abundant protein; IUP = intrinsically unfolded protein)**.

One thousand six hundred and eighty four KEGG orthology assignments were inferred by searching for *P. superbus *unigenes that have homologs among the default set of manually curated eukaryotic genes in the KEGG database (which contains 26 genomes); similarly 1,412 KEGG assignments specific to the *C. elegans *genome were also inferred (Table 3). KEGG pathways associated with metabolism had the highest representation, with a large number of the *P. superbus *sequences associated with the 'carbohydrate metabolism', 'energy metabolism', 'lipid metabolism' and 'amino acid metabolism' pathways. In the environmental information processing category, 'signal transduction' was highly represented. Other highly represented pathways were found in the genetic information processing category including 'translation' and 'folding, sorting and degradation' and a large number of sequences had KEGG assignments to the human neurodegenerative disease sub-category. Many neurodegenerative diseases are associated with the dysfunction or overload of the protection systems responsible for repairing or degrading damaged proteins and macromolecules [59-61]. Cells exposed to severe water stress experience serious damage to their macromolecules and membranes; proteins lose their structures, become unfolded and aggregate. Thus anhydrobiotic organisms are adapted to survive cellular dehydration by deploying efficient cellular protection and repair systems (Figure 2) and it is likely that some gene products that have roles in anhydrobiotic protection in nematodes may also have human homologs which are required for neural survival. For example the molecular chaperone DJ-1, which is associated with familial Parkinson's disease [62,63], is also upregulated in response to desiccation stress in the anhydrobiotic nematode *Aphelenchus avenae *[64]; and AAvLEA1, a natively unfolded late embryogeneis (LEA) protein which is upregulated in response to desiccation stress in *A. avenae *[65], has been shown *in vitro *to protect complex mixtures of proteins from aggregation [66].

**Table 3:** Summary of KEGG assignments of *P. superbus *unigenes to biochemical pathways

KEGG Pathway Category	KEGG mapping of *P. superbus *transcripts to biochemical pathways	
	***Eukaryotes***	***C. elegans***
**1. *Metabolism***	***474***	***438***
1.1 Carbohydrate Metabolism	113	102
1.2 Energy Metabolism	70	68
1.3 Lipid Metabolism	61	47
1.4 Nucleotide Metabolism	36	33
1.5 Amino Acid Metabolism	76	71
1.6 Metabolism of Other Amino Acids	26	24
1.7 Glycan Biosynthesis and Metabolism	21	19
1.8 Metabolism of Cofactors and Vitamins	22	18
1.9 Biosynthesis of Polyketides and Terpenoids	7	9
1.10 Biosynthesis of Secondary Metabolites	13	14
1.11 Xenobiotics Biodegradation and Metabolism	29	33
**2. *Genetic Information Processing***	***294***	***272***
2.1 Transcription	50	43
2.2 Translation	132	124
2.3 Folding, Sorting and Degradation	91	87
2.4 Replication and Repair	21	18
2.5 RNA Family	0	0
**3. *Environmental Information Processing***	***88***	***62***
3.1 Membrane Transport	4	3
3.2 Signal Transduction	73	53
3.3 Signalling Molecules and Interaction	11	6
**4. *Cellular Processes***	***236***	***163***
4.1 Transport and catabolism	91	73
4.2 Cell Motility	19	10
4.3 Cell Growth and Death	63	39
4.4. Cell Communication	63	41
**5. *Organismal Systems***	***250***	***181***
5.1 Immune System	52	38
5.2 Endocrine System	60	49
5.3 Circulatory System	21	15
5.4 Digestive System	44	31
5.4 Excretory System	15	14
5.5 Nervous System	20	10
5.6 Sensory System	19	11
5.7 Development	10	5
5.8 Environmental Adaptation	9	8
**6. *Human Diseases***	***342***	***296***
6.1 Cancers	63	39
6.2 Immune System Diseases	22	21
6.3 Neurodegenerative Diseases	134	130
6.4 Cardiovascular Diseases	36	33
6.5 Metabolic Diseases	2	3
6.6 Infectious Diseases	85	70
***Total ***	***1684***	***1412***

#### Gene ontology assignments

The Gene Ontology consortium has developed a vocabulary of defined terms that describe gene products in the context of three domains--Biological Process, Molecular Function and Cellular Component in a species-independent manner [58]. The representation of GO terms as found by BLAST searches of the *P. superbus *unigenes against genes in the GO database are presented in Additional file 2. In summary, these representations consist of: Biological Process--3,148 BLAST hits; Molecular Function--1,671 hits; and Cellular Component--1,245 hits. Several of these hits were to gene products whose descriptions indicate roles in anhydrobiotic protection, as discussed in the following section.

### Putative anhdyrobiotic and stress response genes constitutively expressed in unstressed *P. superbus*

When cells suffer severe dehydration metabolism ceases, macromolecules denature, membranes undergo phase changes and fuse with other normally separate membranes. Unlike desiccation sensitive taxa, anhydrobiotes have evolved mechanisms which maintain the structure and integrity of macromolecules and membranes in the absence of water and also during rehydration and revival. Comparative studies of the desiccation tolerance phenotypes of anhydrobiotes show lineage specific differences in the response patterns and biochemical adaptations, which implies that anhydrobiotic phenotypes can be achieved in different taxa by the expression of functionally equivalent molecules. Based on currently available data from nematodes and other anhydrobiotic animals we present a model showing the possible steps involved in the detection and expression of anhydrobiotic protection mechanisms in nematodes (Figure 2). Using GO, KEGG and BLAST description data we have identified components of this model in the *P. superbus *unigene dataset and we have assembled and manually annotated a set of 187 candidate genes whose products may be involved in the anhydrobiotic response of *P. superbus*. This dataset is summarized in Table 4 and presented in full in Additional file 3.

**Table 4:** Putative anhdyrobiotic and stress response genes constitutively expressed by unstressed *Panagrolaimus superbus*.

Description	Number of Clusters	Number of ESTs
***Signal Transduction***		
Mitogen-activated protein kinases	3	4
Serine/threonine protein kinases	12	15
Casein kinases	10	15
Protein tyrosine kinases	6	7
Other protein kinases	4	4
Transcription factors/activators	6	9
	***41***	***54***
***Antioxidant Activity***		
Manganese superoxide dismutase (*sod-2*)	1	2
Glutathione peroxidase	3	8
Peroxiredoxin	2	4
Glutathione S-transferase	7	11
Glutaredoxin	2	2
Thioredoxin	1	1
Aldehyde dehydrogenase	2	4
Aldo/keto reductase	2	2
NADP Isocitrate dehydrogenase	1	1
	***21***	***35***
***Late Embryogenesis Abundant Proteins (LEA)***	***13***	***34***
***Heat Shock Proteins (HSP)***		
HSP90 family	3	10
HSP70 family	6	13
HSP60 family	1	1
HSP40/DNaJ family	9	14
Small heat shock protein/α-crystallin family	4	6
HSP90 co-chaperone Cdc37	1	1
HSP70 co-chaperone BAG1	1	2
Tetratricopeptide repeat containing protein	1	1
	***26***	***48***
***Other Chaperone/Chaperonin Proteins***		
Mitochondrial chaperone BCS1 family member	1	1
Mitochondrial prohibitin complex protein 2	1	1
Protein disulfide isomerase	3	9
Cyclophylin family member	5	7
Derlin-2	1	1
DJ-1 family protein	1	2
Prefoldin subunit	2	3
Cytosolic T-complex protein 1	2	3
Putative α-tubulin folding cofactor B	1	1
	***17***	***28***
***The Ubiquitin System***		
Ubiquitin family protein	8	16
Ubiquitin-conjugating enzyme E1	2	2
Ubiquitin-conjugating enzyme E2	5	6
E3 Ubiquitin ligase	5	9
Ubiquitin elongating factor E4	1	1
Ubiquitin carboxyl-terminal hydrolase	2	2
Ubiquitin fusion degradation protein UFD1	1	1
	***25***	***38***
***The Proteasome***		
Proteasome subunit alpha family	4	6
Proteasome subunit beta family	4	6
Proteasome regulatory subunit family	15	24
	***23***	***36***
***Autophagy***		
Autophagy-related protein 2-like (*atg2*)	1	1
LC3, GABARAP AND GATE-16 family member (*lgg-1*)	1	1
	***2***	***2***
***DNA Damage Response Proteins***	***12***	***12***
***Others***		
Aquaporin related family member	2	2
Ezrin/Radixin/Moesin family member (*erm-1*)	2	2
Thaumatin family member (*thn-3*)	1	1
AN1-like Zinc finger family protein	1	1
RIC1 Putative stress responsive protein	1	1
Mitochondrial Lon protease	1	1
	***8***	***8***
***Total***	***187***	***294***

The proposed functions of the proteins encoded by these genes are based on GO, KEGG and BLAST description data. This dataset is presented in full in Additional file 2

#### Signal transduction, protein kinases and transcription factors

Transduction of environmental stress signals is achieved in eukaryotes through a conserved cascade of sequentially acting stress activated protein kinases (SAPKs) which form a branch of the mitogen-activated kinase (MAP-kinase) system [67-70]. In *Saccharomyces cerevisiae *the SAPK pathway is activated by osmostress and the terminal kinase Hog1 [71], when phosphorylated, translocates to the nucleus [72]. Here it phosphorylates several transcription factors, and associates at stress-responsive promoters through such transcription factors [73], resulting in the expression of osmotic response genes. Our *P. superbus *EST dataset contains 35 protein kinases. Among these were three contigs encoding MAP kinases. One of these PSC00478, is a homolog of MAPKAP kinase-2 which is responsible for the phosphorylation of the small heat-shock proteins Hsp27 [74] and α-B-crystallin [75]. The phosphorylation of these small HSPs in response to stresses such as heat shock and oxidation is proposed to regulate actin filament dynamics and to stabilize microfilaments [76]. Two unigenes encode members of the STE20 family of serine/threonine kinases [77]. One of these, PSC03670, has high BLAST identity (3e^-51^) to the *gck-3 *gene whose function is required by *C. elegans *for volume recovery and survival after exposure to extreme hypertonic stress [78]. Nine unigenes encode putative casein kinases, important regulatory molecules in cell division and differentiation and in DNA damage repair [79,80]. Casein kinase 2 (CK2) was found to be upregulated in response to desiccation stress in the nematode *Steinernema feltiae *[81]. Other *P. superbus *stress-related kinases include a homolog of *akt-1*, a regulatory component within the insulin/IGF-1 signaling pathway [82,83], which plays an important role in regulating nematode life span, dauer formation and stress tolerance and diacetylglycerol (DAG) kinase, which modulates DAG levels in the cell membrane, regulating intracellular signalling proteins that have evolved the ability to bind this lipid [84]. DAG kinase is also activated in plants during cold and osmotic stress [85].

Several *P. superbus *unigenes are predicted to encode transcription factors. Among these are a forkhead protein, a member of a conserved family of transcriptional regulators of cellular processes including metabolism, ageing, apoptosis, cell cycle progression and stress resistance [86,87]. Two unigenes encode putative jumonji (JmjC) domain-containing proteins. The *C. elegans jmjc-1 *gene functions as a transcriptional activator of stress related genes in response to multiple stimuli including heat-shock and osmotic and oxidative stress [88]. Two unigenes encode putative high mobility group (HMG) proteins, which are important in modulating chromatin structure and gene expression. HMG transcripts are upregulated in response to desiccation osmotic heat and cold stresses in the anhydrobiotic nematode *Aphelenchus avenae *[64] and HMG function is required for the transcription of stress-responsive genes in *Arabidopsis thaliana *[89].

#### Anti-oxidant activity

Reactive oxygen species (ROS) accumulate in cells as a result of cellular dehydration [90,91]. ROS cause oxidative damage to proteins, lipids, DNA and other macromolecules. Therefore proteins with antioxidant properties are required to rapidly neutralise ROS immediately they are formed. We have characterized 21 unigenes encoding proteins that fall into this category: PSC00113 encodes a manganese superoxide dismutase responsible for converting O_2_^•- ^radicals into H_2_O_2_; three unigenes encode glutathione peroxidases which function to reduce H_2_O_2_; and sequences encoding both the 1-Cys and 2-Cys class of peroxiredoxin enzymes (whose main function is the reduction of peroxides [92]) were also identified.

The tripeptide glutathione (GSH) functions as a co-factor for the antioxidant enzymes glutaredoxin (Grx) and glutathione S-transferase (GST). GSTs catalyse the conjugation of glutathione to reactive electrophilic compounds from endogenous and xenobiotic sources and are thus capable of detoxifying a large variety of cytotoxic molecules [93]. The *P. superbus *dataset contains seven GST unigenes, four from the sigma class of cytosolic GSTs and two from the kappa class of mitochondrial GSTs. The GST sigma and kappa classes are considered to be involved in protection against endogenously produced ROS [94]. PSC02300 encodes a Grx enzyme. Protein deglutathionylation is carried out by Grx [95], and this enzyme can also reduce the disulphide bridges of oxidised proteins [96]. Such disulphide bridges can also be reduced by thioredoxin (PSC00712), which shares a similar structure and overlapping function with Grx [97]. Other *P. superbus *unigenes whose gene products are likely to be involved in antioxidant activity and redox regulation include: aldehyde dehydrogenase which deactivates malondialdehyde, an important end product of lipid peroxidation; two aldo-keto reductase sequences [98,99] and a putative cytosolic NADP-isocitrate dehydrogenase (NADP-ICDH). NADP-ICDH catalyzes the production of NADPH and, by supplying NADPH to the antioxidant systems, NADP-ICDH is an important component in the control of redox balance and the modulation of oxidative damage in the cytosol [100,101].

#### Late embryogenesis abundant proteins

Thirteen *P. superbus *unigenes encode predicted late embryogenesis abundant (LEA) proteins (Table 5). Although LEA proteins have been shown to accumulate during the onset of desiccation in anhydrobiotic animals including nematodes [65,81,102], genes encoding LEA proteins are particularly numerous and heterogeneous in plant genomes. For example the *Arabidopsis thaliana *genome contains 51 *lea *genes placed into 9 different Pfam groups [103,104]. In animal genomes *lea *genes are less abundant and predominantly belong to the Group 3 LEA family (Pfam F02987) [105], with members of LEA Group 1 (PF00477) being described to date only in the brine shrimp *Artemia franciscana *[106] and in an unspecified tardigrade species [107]. Group 3 LEA proteins are highly hydrophilic and largely lacking in secondary structure when fully hydrated [105]. Group 3 proteins also contain blocks of tandemly repeated 11-mer amino acid motifs [108], the number of repeats per LEA protein typically ranging in number from 5 to 24 [109].

**Table 5:** BLASTX similarity searches of *P. superbus *unigenes predicted to encode late embryogenesis abundant (LEA) proteins against the NCBI nr and LEAP [http://forge.info.univ-angers.fr/~gh/Leadb/index.php] databases

Contig ID & [No of ESTs]	*Best BLAST *hit *& [Organism]*	***Database ***&***Accession Number***		BLAST* Bit Score E value		*Disordered AAs (%)*	*GRAVY Index^#^*
PSC00061 [10]	LEA protein [*Caenorhabditis briggsae*]	NCBI nr	CAP25449	82.8	4e^-14^	86%	-0.765
PSC00061	LEA protein [*Arabidopsis thaliana*]	LEAPdb	AAL59922	74.3	8e^-16^		
PSC00416 [6]	LEA protein K08H10.1e [*Caenorhabditis elegans*]	NCBI nr	CCA65580	60.8	1e^-7^	18%	-0.618
PSC00489	LEA protein [*Caenorhabditis elegans*]	LEAPdb	AAB69446	80.5	9e^-18^		
PSC00489 [2]	LEA3 protein [*Glycine max*]	NCBI nr	CAA80491.1	68.6	2e^-10^	10%	-0.810
PSC00416	LEA protein [*Caenorhabditis elegans*]	LEAPdb	AAB69446	74.7	3e^-16^		
PSC00514 [2]	LEA3 protein [*Glycine max*]	NCBI nr	CAA80491.1	64.7	5e^-12^	9%	-0.854
PSC00514	LEA protein [*Pisum sativum*]	LEAPdb	CAF32327	94.0	9e^-22^		
PSC00782 [2]	Hypothetical protein [*Caenorhabditis briggsae*]	NCBI nr	CAP25465	66.2	9e^-12^	100%	-1.367
PSC00782	LEA-like protein [*Arabidopsis thaliana*]	LEAPdb	BAB10116	67.4	4e^-22^		
PSC01414 [1]	LEA protein [*C. briggsae*]	NCBI nr	XP_002637990.1	84.7	5e^-15^	100%	-1.424
PSC01414	LEA-like protein [*A. thaliana*]	LEAPdb	BAD43695	76.6	1e^-16^		
PSC01455 [2]	Hypothetical protein NCU01912 [*Neurospora crassa*]	NCBI nr	XP_965543.1	62.0	7e^-8^	99%	-1.034
PSC01455	LEA-like protein [*A. thaliana*]	LEAPdb	NP_193834	61.2	6e^-12^		
PSC01720 [1]	Predicted protein Gls24 [*Gemella haemolysans*]	NCBI nr	ZP_04776234.1	78.2	8e^-13^	40%	-0.623
PSC01720	LEA-like protein [*A. thaliana*]	LEAPdb	BAD43695	90.5	9e^-21^		
PSC01853 [1]	Hypothetical protein [*Haemophilus influenzae*]	NCBI nr	ZP_01786547	67.0	1e^-9^	100%	-0.988
PSC01853	Hypothetical protein [*H. influenzae*]	LEAPdb	ZP_01786547	67.0		9e^-14^	
PSC03871 [2]	Hypothetical protein K08H10.1f [*C. elegans*]	NCBI nr	CCA65610	42.4	3e^-7^	77%	-0.656
PSC03871	LEA group 3 protein [*Lindernia brevidens*]	LEAPdb	ACA49509	54.7	5e^-10^		
PSC04142 [1]	Hypothetical protein [*Toxoplasma gondii*]	NCBI nr	EEE21041	55.8	2e^-6^	100%	-1.340
PSC04142	LEA-like protein [*A. thaliana*]	LEAPdb	BAD43695	50.4	4e^-09^		
PSC04118 [1]	Protein At5g44310 [*A. thaliana*]	NCBI nr	AAS49101^§^	47.0	1e^-5^	100%	-1.405
PSC04118	LEA-like protein [*A. thaliana*]	LEAPdB	BAD43695	47.0	2e^-9^		
PSC04695 [2]	Hypothetical protein K08H10.1f [*C. elegans*]	NCBI nr	CCA65610	73.6	1e^-11^	100%	-1.297
PSC04695	LEA-like protein [*A. thaliana*]	LEAPdb	BAD43695	67.4	7e^-14^		

The higher e-values against the LEAPdb reflect the smaller number of proteins in this database compared to the NCBI nr database

* These BLASTX searches were carried out using the Blosum 62 substitution matrix

^¶^% Disordered AAs: AA residues with an IUPRED score above 0.5 can be regarded as disordered [110]

**#**Grand average of hydropathicity [111]

^§^The accession numbers BAD43695.1 and AAS49101.1 both correspond to the *A. thaliana *gene At5g44310

A database of LEA proteins has been established recently [112]. Although 89% of the sequences in this database are from land plants, the LEAPdb database includes LEA sequences from animal taxa. Among BLAST searches against the LEAPdb (Table 5), ten *P. superbus *unigenes had best hits to LEA3 proteins from plant species, one unigene was most similar to a putative LEA protein from *Haemophilus influenzae *[113] and two to an LEA3 protein from *C. elegans*, the abundance of *P. superbus *sequences which had hits to plant genes may be a consequence of the large number of plant LEA sequences represented in the LEAP database. The LEA sequences encoded by six of the *P. superbus *unigenes are predicted to be 100% natively unfolded. Three of the 13 *P. superbus *LEA sequences are predicted to lack substantial regions of unfolded structure, however all 13 LEA sequences had negative GRAVY (Grand Average of Hydropathy) indices characteristic of hydrophilic proteins (Table 5). All the predicted sequences showed evidence of tandemly repeated 11-mer amino acid motifs (See Additional file 4). The *C. elegans *genome has been reported to contain three *lea *genes [109] and four *lea *genes have been detected in the *C. briggsae *genome [109]. The best characterized nematode LEA protein is AAv1 which is upregulated in the nematode *A. avenae *in response to desiccation. AAv1 has been shown to protect complex mixtures of proteins from aggregation *in vitro *and *in vivo *[66]. It is possible that some of the *P. superbus *unigenes reported here may represent alternatively spliced forms of a single *lea *gene. However, the relative abundance of *lea *genes in *P. superbus *as compared to *C. elegans*, along with their constitutive expression (we detected 34 LEA-encoding ESTs), suggest that LEA group 3 proteins are an important component of the anhydrobioitc protection repertoire of *P. superbus*.

#### Molecular chaperones and unfolded protein response

Heat shock proteins (HSPs) are essential for the correct folding and maturation of a great diversity of client proteins and for protecting proteins from stress induced unfolding and aggregation [114,115]. Eukaryotic HSP families contain multiple genes, which may be either constitutively expressed or stress inducible and targeted to specific cellular compartments [116,117]. The HSP expression repertoire of an anhydrobiotic organism may thus be very important in maintaining the integrity of the proteome during the dehydration and recovery phases of anhydrobiosis [118-121]. The *P. superbus *dataset contains representatives of all the heat shock protein (HSP) classes characteristic of nematodes, including four distinct small heat shock proteins (sHSP). sHSP are the major "holding" chaperones, retaining unfolding proteins in a conformation suitable for subsequent refolding thus preventing their irreversible aggregation [122,123]. Anhydrobiotic encysted larvae of the brine shrimp *Artemia franciscana *accumulate large quantities of a sHSP known as p26 which constitutes ~15% of the non-yolk protein in these larvae [124]. *A. franciscana *cysts are resistant to desiccation, high temperature, γ-irrradiation and anoxia and the chaperoning activity of p26 is likely to be a very significant component of this remarkable stress resistance [125].

The accumulation of unfolded proteins in the endoplasmic reticulum (ER) arising from physiological or abiotic stress leads to the expression of several protein folding chaperones, including members of the HSP90 and HSP70 families and their co-chaperones [126]. Unfolded protein response (UPR) chaperones from the *P. superbus *dataset include three protein disulfide isomerases (PDI), which catalyse the formation and isomerization (rearrangement) of cysteine bonds during protein folding [127,128]; five cyclophilin-type peptidyl-prolyl cis-trans isomerases which catalyse the isomerisation of the peptide bonds preceding proline residues; and a homolog of Derlin-2 which is required for the degradation of misfolded glycoproteins in the ER [129]. Five *P. superbus *unigenes encode proteins required for the facilitated folding of actin and tubulin to form microtubules: two prefoldin subunits, two subunits of cytosolic T-complex protein 1 and α-tubulin folding cofactor B (Additional file 3). Changes in microtubule dynamics have been shown to occur during osmotic stress in *Zea mays *[130] and during desiccation in *Brassica napus *[131] and it is possible that adjustments to the stability of the microtubule cytoskeleton are also required by *P. superbus *for successful entry into anhydrobiosis.

#### Removal of damaged proteins--the ubiquitin-proteasome (UPS) and autophagy systems

When the HSP chaperone system fails to correctly fold a denatured protein, the misfolded protein is polyubiquitinated. The 26S proteasome, a large multiprotein complex, then translocates polyubiquitinated proteins into the inner proteolytic chamber where they are hydrolysed [132]. If the generation of misfolded proteins exceeds the proteolytic capacity of the ubiquitin proteasomal system (UPS), misfolded proteins accumulate into aggregates which are degraded by autophagy [133,134]. A whole genome RNA interference (RNAi) screen in *C. elegans *identified 40 genes that are essential for survival during acute hypertonic stress [135]. Half of these genes encode proteins that function to detect, transport, and degrade damaged proteins. The importance of the proteasomal system to unstressed nematodes is also apparent from its abundant representation in the *P. superbus *EST dataset, which contains 44 UPS unigenes comprising 68 ESTs (Additional file 3). In contrast, autophagy genes are not well represented in unstressed *P. superbus*. We detected only two *P. superbus *homologs of the 19 core *C. elegans *autophagy genes [136].

#### DNA damage response proteins

DNA extracted from anhydrobiotic stages of the plant parasitic nematode *Ditylenchus dipsaci *was intact, showing no increase in the frequency of double strand DNA breaks (DSBs) as compared with hydrated worms [137]. Data from the anhydrobiotic chironomid *P. vanderplanki *[138] and anhydrobiotic tardigrades [139,140] show that DSBs accumulate with time in the dry state in these organisms. DSBs also accumulate during desiccation in the anhydrobiotic and radiation resistant bacterium *Deinococcus radiodurans*. Similar to *P. vanderplanki *[138] and anhydrobiotic tardigrades [139,140], *D. radiodurans *has acquired the ability to rapidly repair DNA damage when rehydrated [141]. The *P. superbus *dataset contains 12 unigenes encoding proteins involved in DNA repair (Additional file 3). However these DNA repair proteins appear to be constitutively expressed at low levels in *P. superbus*, as each is represented by only a single EST.

#### Other putative anhydrobiotic genes

Other transcripts whose products may play a role in the anhydrobiotic response of *P. superbus *include two putative aquaporins; an *erm *(ezrin, radixin, and moesin) family member; an *an1*-like Zinc finger sequence; a thaumatin-like transcript; two copies of *lon-1 *which encodes a protease that selectively degrades oxidized mitochondrial proteins [142] and a homolog of the *Ric1 *family [143] which encodes plasma membrane proteins that are expressed in response to high salt or low temperature conditions in plants [144]. ERM proteins are activated by osmotic shrinkage [145] and they are thought to function as cross-linkers between plasma membranes and actin-based cytoskeletons [146]. Thaumatin-like proteins are induced in plants in response to pathogens, cold, drought and osmotic stress [147]. The *an1*-like multigene family is involved in plant abiotic stress responses and in inflammation responses in mammals [148].

### A comparison the *P. superbus *EST unigene dataset with EST datasets from other anhydrobiotic nematodes

The 3,982 *P. superbus *unigenes were compared to EST unigene datasets from three other species of anhydrobiotic nematodes [31,102,149] to identify putative homologous protein families which may reveal some of the core anhydrobiotic processes shared by these nematodes. *Plectus murrayi *is an Antarctic soil nematode adapted to survive desiccation and freezing [102]. *Aphelenchus avenae *is a slow desiccation strategist soil dwelling fungiverous nematode. *Ditylenchus africanus *is an endoparasite of plants with peanut as its primary host. It migrates to the pods and seeds of the ground nut and can survive in an anhydrobiotic state in the seeds [149]. The phylogenetic relationships of these nematodes are indicated in Figure 1.

**Figure 3:**
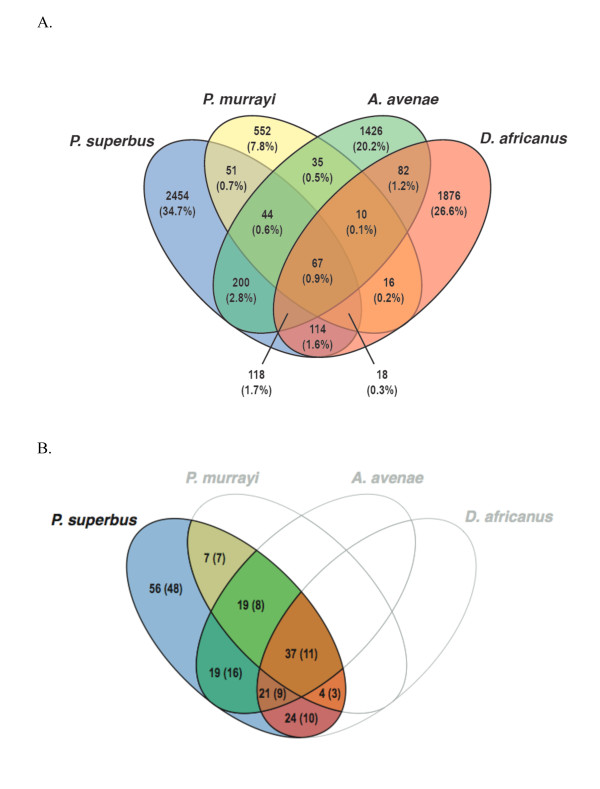
**(a)-Venn diagram indicating the number of unigene families that contain representatives from one or more anhydrobiotic nematodes: *Panagrolaimus superbus, Plectus murrayi, Ditylenchus africanus, Aphelenchus avenae***. The total dataset comprises 7,063 families 67 of which have representative ESTs from all four nematode species. The values in parentheses represent the percentage of the total number of families represented in each section. **(b)-Venn diagram showing the distribution of homologs of the *P. superbus *putative stress response genes from Table **4 **across four anhydrobiotic nematode species**. The values represent the number of *P. superbus *unigenes that have BLAST hits to sequences from one or more of the nematodes *Plectus murrayi, Ditylenchus africanus, Aphelenchus avenae*. The numbers in parentheses correspond to the number of families (see Methods) these unigenes constitute.

The combined dataset from the four anhydrobiotic nematodes comprised 10,791 unigenes. All against all BLASTP analyses of the predicted peptide sequences for these unigenes, followed by their classification into putative homologous groups using the TRIBE Markov clustering algorithm as implemented in the MCL software package [150], has identified 7,063 unigene families, where 6,308 consist of singletons. The distribution of these unigene families across the four nematode species is summarized in Figure 3(a). A total of 67 unigene families contain transcripts from all four anhydrobiotic nematodes. While our analysis is based on an incomplete coverage of the transcriptomes of all four nematodes, these 67 families provide a first indication of subsets of genes common to the four species, some of which may be involved in anhydrobiotic processes. The annotation of the 67 unigene families (inferred by BLAST), together with a list of their component *P. superbus *contigs is presented in Additional file 5. These families include representatives of several of the anhydrobiotic and stress response proteins discussed in the previous section. Among these are protein kinases and HMG proteins; glutathione S-transferase; sHSP, HSP70; HSP90; peptidyl-prolyl cis-trans isomerase; several components of the UPS system and RIC1, a poorly characterized family which encodes plasma membrane proteins that are expressed in response to high salt or low temperature conditions in plants [144]. Members of the nematode specific transthyretin-related (*ttr*) family [151] are also included among the 67 unigenes. The function of *ttr *genes remains elusive; so far the function of just one nematode *ttr *gene product has been discovered (TTR-52 mediates the recognition and engulfment of apoptotic cells in *C. elegans *[152]). Figure 3(b) shows the distribution of homologs of the *P. superbus *putative stress response genes from Table 4 across the four anhydrobiotic nematode species. These BLAST annotations are presented in full in Additional file 3. Since this analysis is based on partial transcriptomes of the four nematodes the results need to be interpreted conservatively, however the data show that these four anhydrobiotic nematodes express a great diversity of stress responsive genes. Surprisingly none of the 13 LEA unigenes were common to all four nematode datsets, and 8 LEA sequences were found only in *P. superbus*. This may indicate that constitutive expression of LEA transcripts is higher in *P. superbus *than in the three other anhydrobiotic nematodes. When more complete coverage of the transcriptomes of anhydrobiotic nematodes and other anhydrobiotic animals becomes available comparative transcriptomic analyses will be a powerful tool for the identification of candidate genes and processes required for successful anhydrobiotic survival.

### Analysis of novel ESTs

**Figure 4:**
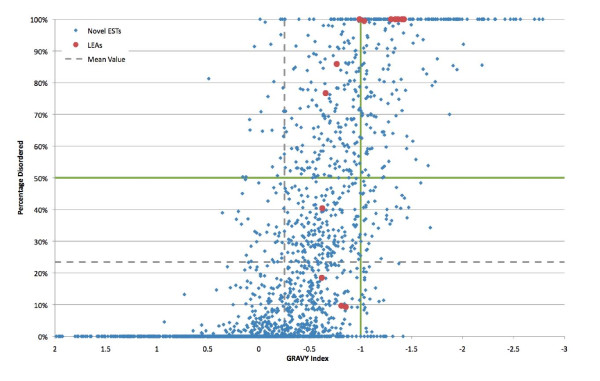
**A plot of the hydropathy value (GRAVY Index) **[**111**]**of *P. superbus *putative novel peptides and the 13 predicted *P. superbus *LEA proteins against their predicted degree of disorder, as determined by the IUPred program **[**110**]. (GRAVY = Grand average of hydropathicity); hydrophilic proteins typically have hydropathy values < -1 [154]. Green lines represent the boundaries that delimit a group of novel hydrophilic peptides that are predominantly disordered.

Of the 3,982 unigenes in our dataset 2,059 (51.7%) have no significant similarity to any sequences in the Genbank or NemPep databases. The Prot4EST algorithm [46] was used to translate these novel unigenes into putative peptides. Analysis of the physical properties of these putative peptides reveals that 149 of them are predicted to lack a fixed tertiary structure (100% intrinsically disordered), while an additional 296 peptides are predicted to be 50-99% disordered. Intrinsically disordered proteins (IDPs) are hydrophilic, being characterized by a high proportion of polar and charged amino acids and low sequence complexity; they also have a low content of the hydrophobic amino acids which would normally form the core of a folded globular protein [153]. These physical features also occur in LEA proteins. A plot the hydropathy [111] of *P. superbus *putative novel peptides and the 13 predicted *P. superbus *LEA proteins against their predicted degree of disorder (determined using the IUPred program [110]) shows that there are 225 *P. superbus *peptides with a GRAVY (Grand Average of Hydropathy) value of ≤ -1 and > 50% disordered (Figure 4).

**Figure 5:**
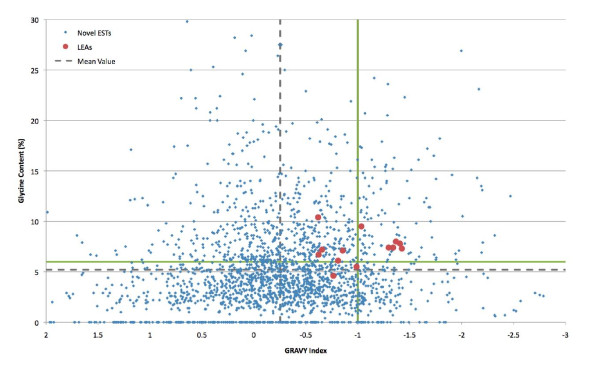
**Plot of the putative glycine content and the hydropathy of the protein sequences encoded by the novel ESTs and putative LEA proteins in the *P. superbus *dataset**. Green lines represent the boundaries of the properties that define hydrophilins; glycine content > 6% and a hydropathicity index of < -1 [154].

Garay-Arroyo *et al. *[154] proposed that LEA proteins are contained within a larger group of proteins called 'hydrophilins' that accumulate in response to osmotic stress in prokaryotes and eukaryotes. The characteristics that define this group are a glycine content of greater than 6% and hydropathy index of less than -1. Our dataset contains 170 novel putative peptides that meet these criteria (Figure 5). The *P. superbus *unigenes predicted to encode LEA proteins (Table 5) were identified on the basis of BLAST searches. Analysis of their physical properties reveals that all of these putative LEA proteins are hydrophilic, having GRAVY values ranging from -0.62 to -1.42; six are predicted to be 100% unstructured, a further three are largely (77-99%) unstructured, but three putative LEA unigenes: PSC01853, PSC00514 and PSC00416 are predicted to be only partly disordered (9, 10 and 18% disorder, respectively). Eleven of the 13 putative LEA sequences also have a glycine content of greater than 6%.

Many IDPs proteins function by molecular recognition: either by transient, or permanent binding to a structured partner molecule [155]. However the functions of some IDPs depend directly on the extended random coil conformation of the disordered state--the so-called entropic chain effect [156]. Entropic chain effects are likely to be central to many of the functions of LEA proteins. The elongated, natively unfolded conformation of LEA proteins may help to form a "molecular shield" [66], preventing protein aggregation and denaturation. These hydrophilic, proteins also have the capacity to bind and retain water molecules and, at later stages of dehydration process, an abundance of charged amino acids may enable some LEA proteins to replace water at the hydrogen bonding sites of dehydrated proteins. Although LEA proteins are natively unfolded when fully hydrated, some LEA proteins, including AavLEA1 [157], have been shown to develop secondary structure as they become desiccated [158], leading to the suggestion that some LEA proteins might function as intracellular space-filler molecules which prevent the collapse of cells as they become desiccated [105]. The combined group of putative hydrophilic proteins identified in Figures 3 and 4 contains 294 individual novel sequences. These sequences represent an important group of candidate anhydrobiotic genes that merit further investigation.

### Expression of putative stress related genes upon desiccation

**Figure 6:**
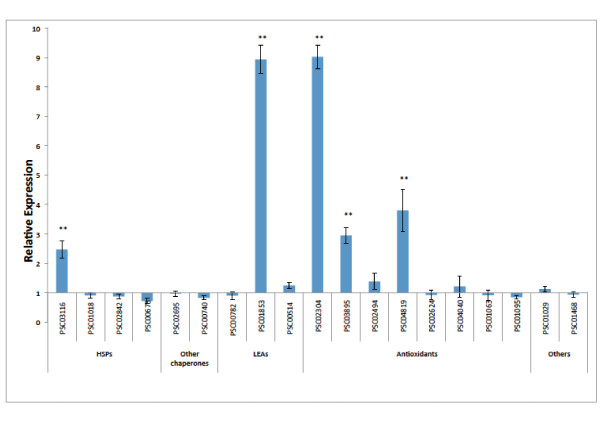
**Real-Time Relative qPCR analysis of the expression of some putative stress-response genes in *Panagrolaimus superbus *following 12 h of desiccation at 98% RH**. The following transcripts were tested: PSC03116: small heat shock protein (sHSP) family member; PSC01018 sHSP family member; PSC02842 HSP40/DNaJ protein family member; PSC00673 HSP70 family member; PSC02695 cyclophilin family member; PSC00740 protein disulfide isomerase; PSC00782 LEA3 protein; PSC01853 putative LEA3 protein; PSC00514 LEA3 protein; PSC02304 DJ-1; PSC03895 1-Cys peroxiredoxin; PSC02494 gluthatione peroxidase; PSC04819 gluthatione peroxidase; PSC02624 glutathione S-transferase (sigma class); PSC04040 glutathione S-transferase (kappa class); PSC01063 aldehyde dehydrogenase; PSC01095 aldehyde dehydrogenase; PSC01029 aquaporin; PSC01468; RIC1 putative stress responsive protein. The reference genes were the *P. superbus 60S ribosomal protein L32 *and *ama-1 *genes. Statistically significant differences (Student's *t *test) are indicated, **p < 0.001.

*P. superbus *is capable of surviving exposure to a dry atmosphere in desiccation chambers containing silica gel without the need for prior preincubation to mild desiccation stress [15]. However it likely that in its natural habitat *P. superbus *would experience more gradual change from a condition in which its cells and tissues are fully hydrated to one of extreme dehydration. In addition, intrinsic behavioural (coiling/clumping) responses or morphological adaptations (such as surface lipids [14] or possibly SXP/RAL-2 cuticular proteins) may slow the rate of water loss in *P. superbus *and allow time for inducible molecular protection mechanisms to be put in place. We used qPCR to investigate the inducible expression of several unigenes that represent homologues of stress related genes in other organisms. The expression of five of the 19 genes tested was upregulated in *P. superbus *following exposure to 98% RH for 12 h (Figure 6).

Three antioxidant genes *gpx *(glutathione peroxidase), *dj-1 *and *prx *(which encodes a 1-Cys peroxiredoxin) were upregulated in response to desiccation stress in *P. superbus*. ROS accumulation is triggered by cellular dehydration and our qPCR data show the importance of enzymatic antioxidant defense systems during the induction of anhydrobiosis. Glutathione peroxidases (GPx) catalyse the reduction of H_2_O_2 _and GPx have been previously found to be upregulated in *A. avenae *and in *P. murrayi *in response to desiccation [64,102]. DJ-1 is a multifunctional protein associated with familial Parkinson's disease [62,63]. One of its proposed functions is a redox-dependent molecular chaperone activity [63] and a role for DJ-1 as an atypical peroxiredoxin-like peroxidase in inactivating mitochondrial H_2_O_2 _has also been proposed [159]. We show that the expression of *dj-1 *is upregulated 9-fold in *P. superbus *in response to desiccation stress. This gene is also upregulated in response to desiccation stress in the anhydrobiotic nematode *Aphelenchus avenae *[64]. Peroxiredoxins (Prx) comprise two classes: 1-Cys Prx and 2-Cys Prx, based on the number of cysteinyl residues directly involved in catalysis [92]. Animal Prx sequences comprise 3 clades [160]: clades A and B contain 2-Cys Prx, while 1-Cys Prx occur in clade C which also contains plant 1-Cys Prx sequences [160]. In plants 1-Cys Prx are seed-specific [161]: they accumulate during seed maturation and their expression declines during germination, an expression pattern is also characteristic of many *lea *genes. A seed-specific 1-Cys Prx, was found to be abundantly expressed during desiccation in the leaves of the resurrection plant *Xerophyta viscosa *[162] and transcripts encoding a 1-Cys Prx are also upregulated during rehydration of the anhydrobiotic moss *Tortula ruralis *[163]. Here we show that a 1-Cys Prx is upregulated in response to desiccation in *P. superbus*, revealing a further parallel between the desiccation tolerance mechanisms of anhydrobiotic nematodes and plants.

Only one of the three *lea *sequences tested was upregulated, but this sequence (PSC01853) was upregulated 9.8 fold in response to desiccation stress. Of the four *P. superbus hsp *sequences assayed only one, an *shsp *sequence, was upregulated in response to desiccation. The genes encoding HSP70 and HSP90 are constitutively expressed in the Antarctic nematode *Plectus murrayi *and are not upregulated further by desiccation [164]. Similarly only 2 of 6 *hsp70 *paralogues show higher expression levels in diapausing eggs of the rotifer *Brachionus plicatilis *than in other metabolically active life stages [165]. sHSP are the major holding chaperones which prevent the irreversible aggregation of unfolding proteins [122,123]. They have been shown to accumulate in anhydrobiotic encysted larvae of the brine shrimp *Artemia franciscana *[124] and they are also abundantly expressed in diapausing eggs of *B. plicatilis *[165]. These expression profiles for representatives of different HSP classes in anhydrobiotic animals from three different phyla suggest that sHSP proteins, in particular, have an important role in maintaining the integrity of the proteome during anhydrobiosis.

## Conclusions

We obtained 9,216 ESTs from an unstressed mixed population of the anhydrobiotic nematode *P. superbus *and we derived 4,009 putative *P. superbus *unigene sequences from these ESTs. The finding that 51% of these unigenes correspond to novel sequences is consistent with previous metagenomic analyses of nematode EST datasets, and is a reflection of the diversity of nematode gene space. Functional annotation of the *P. superbus *unigenes has identified 187 constitutively expressed consensus sequences encoding putative stress-related genes that may have a role in anhydrobiosis. Among these were: MAP-kinases; members of the jumonji family of transcription activators; antioxidant enzymes; molecular chaperones; components of the ubiquitin-proteasome system; DNA damage response proteins and late embryogenesis abundant (LEA) proteins. Thirteen *P. superbus *unigenes encode predicted LEA proteins, all members of Group 3 as is typical of animal LEA proteins. The relative abundance of *lea *genes in *P. superbus *as compared to *C. elegans*, along with their constitutive expression (we detected 34 LEA-encoding ESTs), suggest that LEA proteins are an important component of the anhydrobiotic protection repertoire of *P. superbus *and that the *lea *gene family may have undergone lineage-specific expansion in this species. *P. superbus *appears to utilize a strategy of combined constitutive and inducible gene expression in preparation for entry into anhydrobiosis. Five of the 19 putative *P. superbus *stress response genes tested were upregulated in response to desiccation. Three of the upregulated genes encoded antioxidant enzymes, an indication of the importance of enzymatic antioxidant defense systems during the induction of anhydrobiosis. One of the upregulated genes encoded a 1-Cys Prx, revealing a parallel between the desiccation tolerance mechanisms of plant seeds and resurrection plants with those of anhydrobiotic nematodes. Of the four *P. superbus hsp *sequences assayed only one, an *shsp *sequence, was upregulated in response to desiccation. This is consistent with the expression profiles for representatives of different HSP classes in anhydrobiotic animals from other phyla and suggests that sHSP proteins have an important role in maintaining the integrity of the proteome during the dehydration phases of anhydrobiosis.

A large number of *P. superbus *unigenes are homologous to human disease genes, particularly those implicated in neurodegenerative diseases. Many neurodegenerative diseases are associated with the dysfunction of the protection systems responsible for repairing or degrading damaged proteins and macromolecules, thus some gene products that have roles in anhydrobiotic protection in *P. superbus *may have human homologs that are required for neural survival. So, in addition to providing candidate genes for use in anhydrobiotic engineering experiments, knowledge of the molecular mechanisms responsible for anhydrobiotic protection of macromolecules may also provide insights into some of the gene products required for the integrity of neural tissues.

Analysis of the physical properties of the putative peptides encoded by the 2,059 novel *P. superbus *unigenes reveals that 149 of them are predicted to be 100% intrinsically disordered (IDPs) and that 170 novel sequences meet the criteria used to define 'hydrophilin' molecules which accumulate in response to osmotic stress in prokaryotes and eukaryotes. These IDPs and putative hydrophilins represent a key group of potential stress-related genes. The most highly expressed *P. superbus *sequence belongs to the nematode specific family of SXP/RAL-2 proteins, which had previously been identified as a class of secreted and surface associated antigens in diverse animal parasitic nematodes. The abundant representation of the SXP/RAL-2 in *P. superbus *may indicative of a role for this protein in stabilizing the nematodes' integument and slowing the rate of water loss during evaporative desiccation.

*Panagrolaimus *is an excellent model system for the study of anhydrobiosis and cryobiosis. Panagrolaimid nematodes can be readily cultured in the laboratory and have a short generation time. The anhydrobiotic and cryobiotic species and strains of *Panagrolaimus *described to date belong to a single clade, which will facilitate comparative transcriptomic analyses of the molecular basis of anhydrobiosis in a single genus. This study is the first investigation of the putative molecular mechanisms involved in anhydrobiosis in *Panagrolaimus*. Because of its provenance and its anhydrobiotic and cryotolerant phenotypes the genome of *P. superbus *is currently being sequenced as part of the 959 Nematode Genomes Initative [24]. In addition to providing cDNA clones and sequence data for candidate anhydrobiotic genes, the dataset presented here will provide anchor sequences important for the assembly of the genome and transcriptome of *P. superbus *from high-throughput sequence data.

## Methods

### Nematode culture

*Panagrolaimus superbus *(strain DF5050) was obtained from Prof. Bjorn Sohlenius, Swedish Museum of Natural History, Stockholm. The nematodes were cultured at 20°C in the dark on nematode growth medium (NGM) plates containing a lawn streptomycin resistant *E. coli *strain HB101 obtained from the *Caenorhabditis *Genetics Center, University of Minnesota, USA. The NGM was supplemented with streptomycin sulfate (30 μg ml^-1^).

### cDNA library construction and EST generation

Total RNA was extracted from mixed stage unstressed worms using the TRIzol^® ^reagent (Invitrogen, Carlsbad, USA). The cDNA library was prepared using the SMART™ cDNA Library Construction Kit Long-Distance (LD) PCR protocol (Clontech, Mountain View, CA 94043 USA). Fifty ng of total RNA was used for the SMART cDNA synthesis and there were 25 PCR cycles in the LD PCR amplification step. The cDNAs were cloned into the pDNR-Lib vector (Clontech) and transformed into *E. coli *DH10B cells. A total of 15,360 recombinant *E. coli *were picked using a Q-Bot™ robot (Genetix, Hampshire BH25 5NN, UK) and transferred to 384 well microtitre plates containing freezing media [166] and chloramphenicol (30 μg ml^-1^) and the plates were stored at -80°C. The cDNA inserts from individual transformants (n = 9,216) from the cDNA library were sequenced by the Sanger method at the Scottish Crop Research Institute, Dundee (4,224 clones) and at The GenePool, University of Edinburgh (4,992 clones).

### Clustering and sequence analysis

The raw EST sequences were processed through the PartiGene pipeline [27], first using trace2dbEST which removes vector-derived sequences, poor quality sequences and ESTs shorter than 150 bp, followed by CLOBB [167], an iterative program which groups the sequences on the basis of BLAST similarity into clusters that are putatively derived from the same gene. Clusters containing more than one sequence were then assembled into consensus sequences using phrap (http://www.phrap.org). The partial transcriptome consists of these consensus sequences, along with those clusters that contain only one sequence (singletons). Potential bacterial contaminant sequences (28 contigs) and nematode rRNA genes (27 contigs) were identified using a BLASTN search of the *P. superbus *consensus sequences against the GenBank nucleotide database (nt), with an e-value cut-off of 1e^-50 ^to identify significant matches. Genes encoded on the mitochondrial genome were identified by BLASTN using 27 nematode mtDNA genomes from GenBank as queries against the *P. superbus *consensus sequences with an e-value cut-off of 1e^-10^.

The consensus sequence for each unigene was translated using prot4EST [46]. Each unigene was then subjected to a BLASTP search (e-value cut-off of 1e^-4^) against a non-redundant custom database containing sequences from a variety of sources: the GenBank NR database, Wormpep (version 224) (http://www.sanger.ac.uk/Projects/C_elegans/WORMBASE/current/wormpep.shtml) and an extended version of the Nempep4 database (http://www.nematodes.org/nembase4/), which we have named Nempep4+. Nempep4+ has been supplemented with sequences from the following nematodes: *Plectus murrayi, Ditylenchus africanus, Aphelenchus avenae, Trihinella spiralis, Wuchereria bancrofti, Loa loa *and *Pristionchus pacificus*. Putative genes were annotated using the annot8r algorithm [56]. This software tool assigns Gene Ontology (GO) terms, Enzyme Commission (EC) numbers [168] and Kyoto Encyclopaedia of Genes and Genomes (KEGG) pathway data [57] to EST sequences based on BLAST searches against annotated subsets of the EMBL UniProt database [169]. All BLAST results were parsed and the corresponding annotations were saved in a relational postgreSQL database (http://www.postgresql.org). A web interface where the annot8r annotations can be subjected to keyword queries and where output clusters can retrieved is available at http://www.nematodes.org/nembase4/species_info.php?species=PSC. Supplemental ortholog assignment and pathway mapping were carried out using the KAAS-KEGG Automatic Annotation Server [170].

To identify putative unigene families among four anhydrobiotic nematodes, EST consensus sequences were kindly provided by various groups: 1,387 *Plectus murrayi *sequences [102] from Dr. B. Adhikari; 2,596 *Ditylenchus africanus *sequences [149] from Dr. A. Haegeman and 2,700 *A. avenae *sequences [31] from Dr. N. Karim. All ESTs were translated into peptide sequences using prot4EST [46] and they were subjected to an all-vs-all BLASTP analysis to identify pairwise similarities. A graph representation of the homologous relationships among the unigenes was constructed, where each node is a unigene and an edge is drawn between any two nodes that have a BLASTP match. Each edge is weighted by -logE where E is the e-value of the alignment between two similar unigenes. E-values of 0 are transformed into 1e^-200^, i.e. an edge weight of 200. This graph is then used by the MCL algorithm [150] as input, with an inflation parameter of 2.1, to classify the unigenes into putative families.

### Translation and primary structure analysis of novel ESTs

Novel ESTs were translated into putative peptide sequences using prot4EST which incorporates the ESTScan2.0 [171] and DECODER [172] programs which use '*de novo*' prediction methods for predicting the amino acid sequence of cDNA sequences with putative sequencing errors (particularly insertions and deletions). The glycine content and the Grand Average of Hydropathy (GRAVY) values of these putative peptides were determined using the ProtParam tool available at http://www.expasy.ch/tools/protparam.html[173]. The GRAVY value is calculated as the sum of hydropathy values of all the amino acids, divided by the number of residues in the sequence [111]. Predictions of the extent of intrinsically disordered regions within each putative peptide were determined using the IUPred program (using the long disorder prediction algorithm) [110] kindly provided by Dr. Zsuzsanna Dosztányi.

### Real-time relative qPCR analysis of gene expression

A mixed population of nematodes was vacuum filtered onto 25 mm Supor^® ^Membrane Disc Filters at a concentration of 2,000 nematodes per filter. Five replicate filters were prepared for each treatment. The filters were placed in an 8 L glass desiccation chamber over a saturated solution of potassium dichromate (to generate an RH of 98% [174]) for 12 h at 20°C in the dark. Nematodes were then washed off the filters with distilled water and the nematodes from the five filters were pooled together. RNA was extracted using the TRIsure™ (BIO-38033, Bioline) method followed by treatment with DNAse I (Promega, M6101). Control nematodes were placed directly in to TRIsure and flash frozen with liquid N_2 _without vacuum filtration.

Total RNA (1 μg per reaction) was converted to cDNA using the Roche Transcriptor First Strand cDNA Synthesis Kit (04 379 012 001). One μl of cDNA from the above reaction was used for each real time qPCR reaction. These reactions were carried out on a Roche LightCycler 480 thermocycler using Roche SYBR I Master 1 kit (04 707 516 001). Each qPCR reaction also contained 5 μl SYBR Master Mix, 0.002 pmole of each primer and 2 μl H_2_0. Primers were designed to produce an amplicon of approximately 125 bp for each gene tested. (These primer sequences are presented in Additional file 6). Relative expression data were calculated with the LightCycler 480 Efficiency Method analysis software using the second derivative maximum option. The *P. superbus ama-1 *and *rpl32 *genes were used as a reference. Having established that the crossing point data were normally distributed and that the variance of the controls and treatment data were equal, two sample Student's t-tests were carried out to identify statistically significant differences in expression levels between the controls and the experimental treatments.

### Availability of supporting data

These *P. superbus *EST sequences have been deposited in dbEST with the accession numbers GW405912-GW413517. The unigene sequences and annotations along with their constituent ESTs can be downloaded from the NEMBASE4 database [37] at http://www.nematodes.org/nembase4/species_info.php?species=PSC and the unigene annotations can also be subjected to keyword queries at the NEMBASE4 database.

## Competing interests

The authors declare that they have no competing interests.

## Authors' contributions

MS and JTJ prepared the *P. superbus *cDNA library and sequenced 4,224 cDNA clones. MB co-ordinated the sequencing of 4,992 cDNA clones. MB and TT processed the EST sequences through Partigene. SW, TT, GOM, AB and MP carried out BLAST analyses and sequence annotations. BE did the prot4EST translations and prepared the dataset for inclusion in NEMBASE4. MBD and TT did the ProtParam and IUPred analysis for the novel sequences. GOM carried out the KEGG analyses. SW did the TRIBE MCL analysis. SW, GOM and AB compared the *P. superbus *dataset to that of other anhydrobiotic nematodes. TT and AB analysed the LEA sequences. EDM carried out the qPCR experiments. TT and AB drafted the ms. MB and JTJ provided critical input into the first draft of the ms. AB co-ordinated the project. All authors read and approved the final manuscript.

## Supplementary Material

Additional file 1 **Most abundantly represented novel transcripts in a dataset of 7,606 ESTs prepared from a mixed stage unstressed culture of the anhydrobiotic nematode *Panagrolaimus superbus***. Click here for file

Additional file 2 **The representation of Gene Ontology (GO) terms recovered in BLAST searches of the *P. superbus *unigenes against the GO database**. Click here for file

Additional file 3 **Putative anhydrobiotic and stress response genes constitutively expressed by an unstressed mixed stage population of *Panagrolaimus superbus***. Aa = *Aphelenchus avenae*; Da = *Ditylenchus africanus*; Pm = *Plectus murrayi*. Click here for file

Additional file 4 **An alignment of the *Panagrolaimus superbus *LEA sequence encoded by PSC00061 with a *Caenorabditis briggsae *LEA protein (Accession Number CAP25449)**. This *C. briggsae *sequence was most similar to PSC00061 in a BLASTx search of the NCBI nr database (Table 5). Putative 11-mer repeats are indicated in colour. Click here for file

Additional file 5 **The identities of the 67 TRIBE MCL unigene families which contain transcripts from each the anhydrobiotic nematodes *Panagrolaimus superbus, Aphelenchus avenae, Ditylenchus africanus *and *Plectus murrayi*, along with a list of the component *P. superbus *contigs contained in each family**. Click here for file

Additional file 6 **The primer sequences used for real time qPCR analysis of gene expression in *Panagrolaimus superbus *in response to desiccation stress**. Click here for file
