# Supplementary material for: A molecular analysis of desiccation tolerance mechanisms in the anhydrobiotic nematode Panagrolaimus superbus using expressed sequenced tags
Source: BMC Res Notes. 2012 Jan 26;5:68. doi: 10.1186/1756-0500-5-68 (PMC3296651; doi:10.1186/1756-0500-5-68)
Supplement: Additional file 2 — The representation of Gene Ontology (GO) terms recovered in BLAST searches of the P. superbus unigenes against the GO database. [file 1756-0500-5-68-S2.DOC]

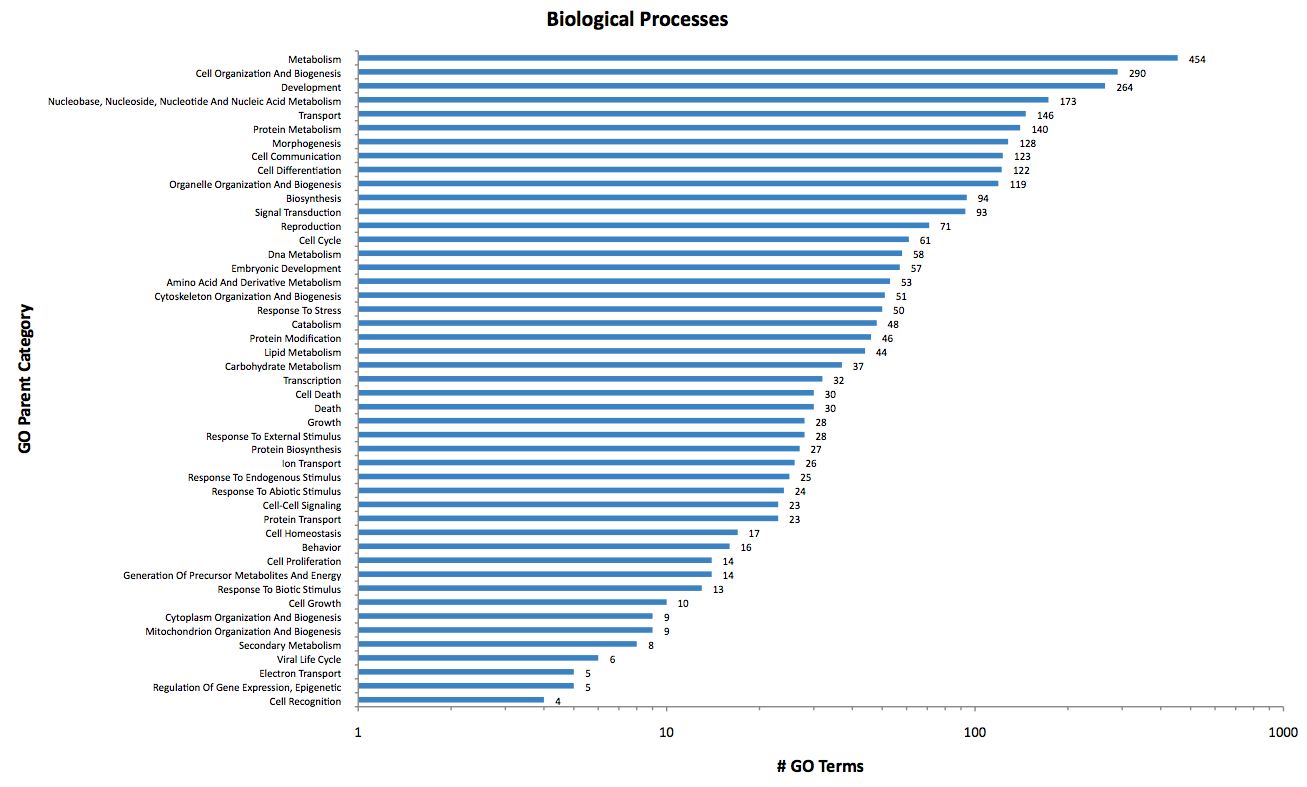


Additional File 2 (a). Representation of *Panagrolaimus* *superbus* unique sequences mapped to Gene Ontology (GO) biological processes category


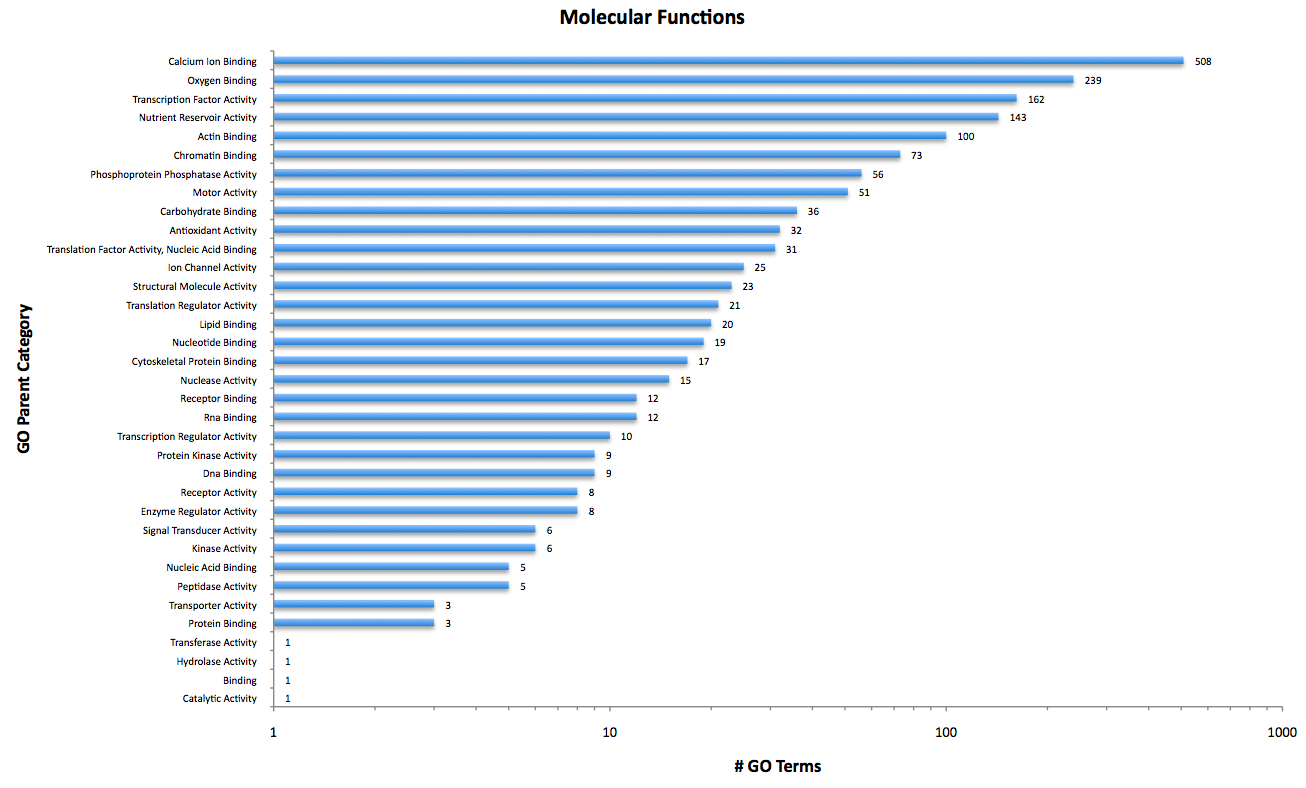


Additional File 2 (b). Representation of *Panagrolaimus* *superbus* unique sequences mapped to Gene Ontology (GO) molecular functions category


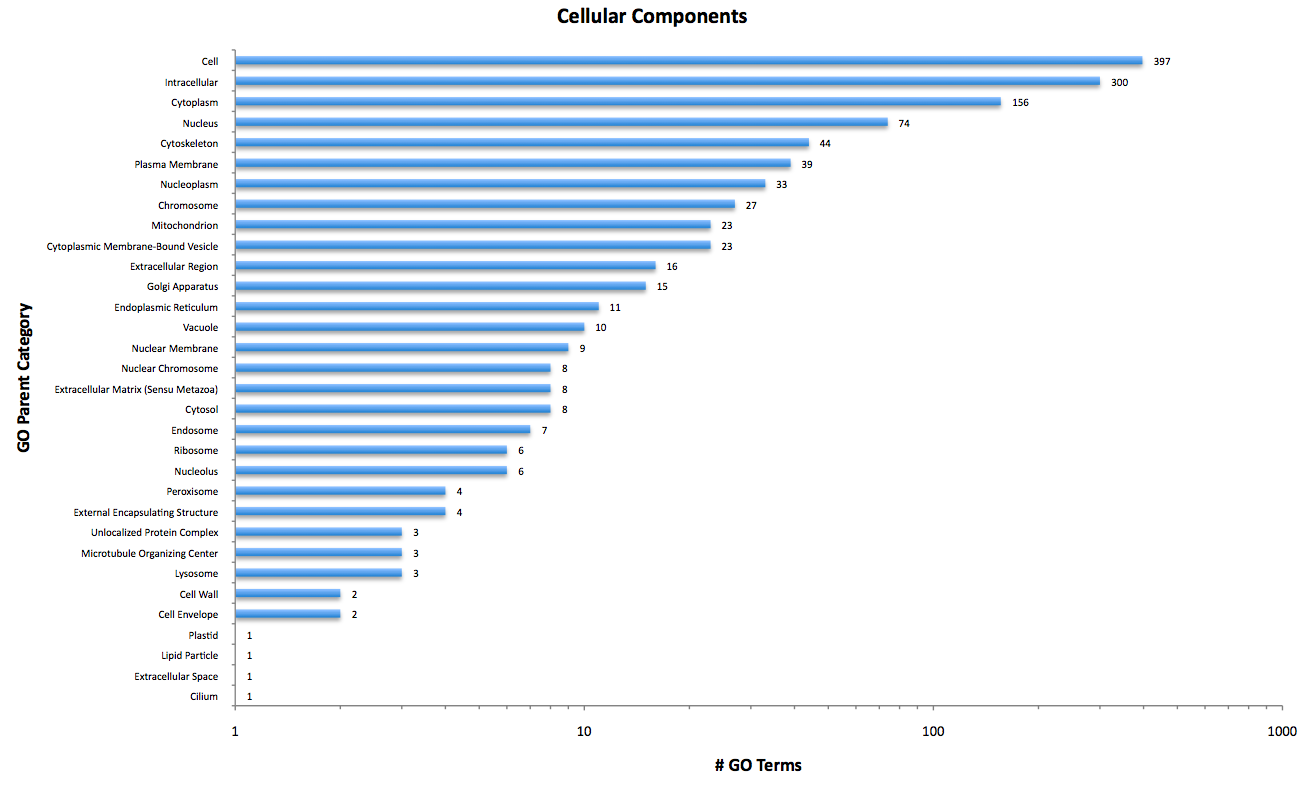


Additional File 2 (c). Representation of *Panagrolaimus* *superbus* unique sequences mapped to Gene Ontology (GO) cellular components category
