# Supplementary material for: A molecular analysis of desiccation tolerance mechanisms in the anhydrobiotic nematode Panagrolaimus superbus using expressed sequenced tags
Source: BMC Res Notes. 2012 Jan 26;5:68. doi: 10.1186/1756-0500-5-68 (PMC3296651; doi:10.1186/1756-0500-5-68)
Supplement: Additional file 4 — An alignment of the Panagrolaimus superbus LEA sequence encoded by PSC00061 with a Caenorabditis briggsae LEA protein (Accession Number CAP25449). This C. briggsae sequence was most similar to PSC00061 in a BLASTx search of the NCBI nr database (Table 5). Putative 11-mer repeats are indicated in colour. [file 1756-0500-5-68-S4.DOC]

PSC00061 ---------------------------- ----------QVKP VVLDTADVVID ASKDALEAAGD

Cb_lea 300 ENKAADTYNTAKYKAADAYDDAKDKAGN AWEATKDKAANAKE AAGDKADEASD KAKSMTEKAGD

:.* .. *.** . * :*. * ***

PSC00061 27 NIYS ATQTTIEKGKE LFGASKEKAAE LADSASQKAGE IKETVAGKANE FADSASNTAQN VV

Cb_lea 365 KISG AWEATKEKAQD VVDSFKGHSTD TKDNVENKAAD MYNSAKDKAGN AWDATKDKAHN AK

:* . * ::* **.:: :..: * :::: *...:**.: : ::. .**.: *::.:.*:* .

PSC00061 87 DKTRENAAA AAD RTKEVAGDAKE AIGE VGEKSRQKAQE VASSAKATAQN TRSSPID----

Cb_lea 426 EAAGDKADE ASD KAKSMTEKAGD KISG AWEATKDKAQD VVDSFKGHATN TKDSAQNKAAD

: : ::* *:* ::*.:: .* : *. . * :::***: *..* *. * * *:.*. :

PSC00061 143 -YSSEKVVENP MNETNRVFVEE KIEVKHERP-- ------------------ LTTTEKVDI--

Cb_lea 486 AYNSAKDAAGD AWDATKDKAEE VKDKAHDKKED YKERCSEAKDRATGQPHG PLETAKDKISG

*.* * . . ::.: .** : *:: *:.:*:.

PSC00061 183 VEHSTVDEGAS FADKVKGHTDI TTDDVHEHTKQ QLHHG------ --------AHQ VKHAANN

Cb_lea 548 AWEATKDKAQD VVDSFKGHSTD AKDNVENKASD MYNSAKDKAGN AWDATKDKAHN AKEVAGD

. .:* *:. . ..*..***: :.*:*.:::.: : . **: .*..*.:

PSC00061 231 AADN LRH NAEA------------

Cb_lea 700 KADE ASD KAKSMTEKAGD KISG

**: . :*::

**Additional File 4 - An alignment of the *Panagrolaimus superbus* LEA sequence encoded by PSC00061 with a *Caenorabditis briggsae* LEA protein (Accession Number CAP25449).** This *C. briggsae* sequence was most similar to PSC00061 in a BLASTx search of the NCBI nr database (Table 5). Putative 11-mer repeats are indicated in colour.
