# Supplementary material for: A molecular analysis of desiccation tolerance mechanisms in the anhydrobiotic nematode Panagrolaimus superbus using expressed sequenced tags
Source: BMC Res Notes. 2012 Jan 26;5:68. doi: 10.1186/1756-0500-5-68 (PMC3296651; doi:10.1186/1756-0500-5-68)
Supplement: Additional file 6 — The primer sequences used for real time qPCR analysis of gene expression in Panagrolaimus superbus in response to desiccation stress. [file 1756-0500-5-68-S6.DOCX]

Additional File 6. The primer sequences used for real time qPCR analysis of gene expression in *Panagrolaimus superbus* in response to desiccation stress.

| ***P. superbus* Cluster ID** | **Description** | **Primer** | **Sequence 5’-> 3’** |
| --- | --- | --- | --- |
| PSC00673 | HSP70 family | PShsp-70F | ACGTGCAATGACCAAAGACA |
|  |  | PShsp-70R | ACCATTGGCATCAACATCAA |
| PSC02842 | HSP40/DNAJ  family member | PShsp-40F | AAACAAGCCGTTGAAGCACT |
|  |  | PShsp-40R | GCAGGCGATACTCCAAGAAC |
| PSC03116 | sHSP  family member | PShsp-12F | ACTCCAACATGGACGGAAAA |
|  |  | PShsp-12R | ACGGTTGCCAATTTGCTATT |
| PSC01018 | sHSP 21 Bursaphelenchus | PShsp-21F | GTTCATTCCTTCGTCGGGTA |
|  |  | PShsp-21R | AGGCTTTGGAGCAAAGATGA |
| PSC00782 | LEA Protein | PSlea-2F | TGGAATCCTCATCTCCAACA |
|  |  | PSlea-2R | GCAGCATCATAGGCATCAGA |
| PSC01853 | LEA Protein | PSlea-5F | GGAGCTGCAAAGGTTAAAGC |
|  |  | PSlea-5R | ATGGCATCTTGTTGTTCACG |
| PSC00514 | LEA Protein | PSlea-8F | GCTGGTAAAGCTAAGGATGTTATG |
|  |  | PSlea-8R | GAACATTATCCCATGTTTCTTCAGC |
| PSC02695 | Cyclophilin family member | PScyp-3F | TATCTGCACTGCCGTTACCA |
|  |  | PScyp-3R | TCGGCAGAAGTTTTTCCACT |
| PSC00740 | Protein disulfide isomerase | PS00740F | GCAAAACTGGAGCTGGTCTC |
|  |  | PS00740R | AAACAGGCAATTTGCGTACA |
| PSC01029 | Aquaporin | PS001029F | TTAGGAAATGCCCTCATTGG |
|  |  | PS001029R | CAAGAACAAGGAAGGCAAGG |
| PSC03895 | Peroxiredoxin 1 | PScys-2F | TGGGGCTTAAACTTGGTGAC |
|  |  | PScys-2R | GTTGTGCAGACAGGCGTAAA |
| PSC01468 | RIC1 (Putative stress responsive protein) | Psric-1F | CCCCGATTATGTTGCTCTGT |
|  |  | Psric-1R | ATCCGGGGATATAACCCAAA |
| PSC02304 | DJ-1 family protein | PSdj-1F | AGCGCCAGTTATTTTTGCAC |
|  |  | PSdj-1R | CCTGGAGCTCGACTCGTTAC |
| PSC02494 | Glutathione peroxidise | PSC02494-F | TGATGATGCAGCACCACTTT |
|  |  | PSC02494-R | TGGAGCGAAACGTTTAACAA |
| PSC04819 | Glutathione peroxidise | PSC04819-F | TCAAGAACCTGCGGAAAATC |
|  |  | PSC04819-R | GCCGTTGACTTCAAGCTTTC |
| PSC02624 | Glutathione S-transferase (sigma class) | PSC02624-F | CCCCAAGAATGATTTTGCAT |
|  |  | PSC02624-R | TTTGCCATCAACTTCAAGGA |
| PSC04040 | Glutathione S-transferase (kappa class) | PSC04040-F | GGAGCTCCATGGTTTGTCAT |
|  |  | PSC04040-R | ATGGGCTCCAACAAAATCAA |
| PSC01063 | Aldehyde dehydrogenase | PSC01063-F | GTTGCACGTCGAATTGTTTG |
|  |  | PSC01063-R | CAAGTTCATCACGCTTTGGA |
| PSC01095 | Aldehyde dehydrogenase | PSC01095-F | TGATTTCGCTGTAGGCCTTT |
|  |  | PSC01095-R | AACCCCAACAACACCAAGAG |
| PSC01944 | RNA polymerase II | PSRNAPOLII-F | GATGACTTTATGGAAGAAGATGAGG |
|  |  | PSRNAPOLII-R | CTATGATCACAATTTCGGCAAG |
| PSC00238 | 60S ribosomal protein L32 | PS60SL32-F | GTTCGTAGACGTTTCAAGGGTACT |
|  |  | PS60SL32-R | TCGAGATCTCTGACATTATTGACG |
